# Multi-Object Tracking Algorithm for RGB-D Images Based on Asymmetric Dual Siamese Networks

**DOI:** 10.3390/s20236745

**Published:** 2020-11-25

**Authors:** Wen-Li Zhang, Kun Yang, Yi-Tao Xin, Ting-Song Zhao

**Affiliations:** Faculty of Information Technology, Beijing University of Technology, Beijing 100124, China; yangkun@emails.bjut.edu.cn (K.Y.); xinyidao@emails.bjut.edu.cn (Y.-T.X.); Zhaotingsong@emails.bjut.edu.cn (T.-S.Z.)

**Keywords:** RGB-D, asymmetric dual Siamese network, multi-object tracking

## Abstract

Currently, intelligent security systems are widely deployed in indoor buildings to ensure the safety of people in shopping malls, banks, train stations, and other indoor buildings. Multi-Object Tracking (MOT), as an important component of intelligent security systems, has received much attention from many researchers in recent years. However, existing multi-objective tracking algorithms still suffer from trajectory drift and interruption problems in crowded scenes, which cannot provide valuable data for managers. In order to solve the above problems, this paper proposes a Multi-Object Tracking algorithm for RGB-D images based on Asymmetric Dual Siamese networks (ADSiamMOT-RGBD). This algorithm combines appearance information from RGB images and target contour information from depth images. Furthermore, the attention module is applied to repress the redundant information in the combined features to overcome the trajectory drift problem. We also propose a trajectory analysis module, which analyzes whether the head movement trajectory is correct in combination with time-context information. It reduces the number of human error trajectories. The experimental results show that the proposed method in this paper has better tracking quality on the MICC, EPFL, and UMdatasets than the previous work.

## 1. Introduction

With the rapid development of modern computer technology, Multi-Object Tracking (MOT) algorithms have received the attention of many research scholars. The main task of the multi-object tracking algorithm is to track and label the trajectory of each target in a scene through a video sequence, which is widely used in indoor security [1,2], video surveillance [3,4], and human-computer interaction [5,6]. Currently, MOT algorithms are mainly used in surveillance systems for indoor public places. They analyze the trajectory of multiple pedestrians in a surveillance scene and provide accurate and stable information for intelligent surveillance systems. It can help managers to make real-time and accurate management decisions. However, MOT algorithms also bear a variety of challenges such as target occlusion and target attitude changes. In order to overcome these challenges, many researchers have improved the existing MOT algorithms to enhance accuracy and stability.

From the data used in the MOT algorithms, most of the algorithms [7,8,9,10,11,12,13,14,15,16,17,18,19,20] use Two-Dimensional (2D) images (RGB images), which are acquired by the visible light camera for analysis and processing. They use RGB images to detect the 2D position of the target. Then, they provide the target’s trajectory based on the target’s appearance information or 2D motion information. However, the RGB images that are acquired by the visible light camera can hardly reflect the position relationship of the various objects in the scene. When a large number of targets is gathered in the scene, it is difficult to use the target’s appearance information or 2D motion information to distinguish the identity of multiple targets in the scene. It is easy to cause the tracking algorithm to switch the target track ID frequently, and these algorithms would likely lead to the problem of track disconnection.

In recent years, the price of depth cameras has become lower and lower with the development of manufacturing technology for depth camera equipment. It has become effective and feasible to use depth cameras for video surveillance systems. Depth images that are acquired by depth cameras can directly reflect the position relationship information and shape contour information among various objects in the scene. Some researchers have fused depth images with RGB images to generate the RGB-D feature of the target. This feature has been applied in many image analysis tasks [21,22,23] with good results.

Compared to RGB images, depth images could effectively separate the foreground and background areas within a scene. They also could highlight the contours of each target in the scene and effectively distinguish between mutually occluded targets. However, depth images do not contain the target’s appearance information, and the algorithm cannot distinguish the identity information of the target. Hence, it is impossible to track the target only using the depth images.

To improve tracking quality, some researchers fused appearance information from RGB images with distance position information from depth images. This overcame the interference of occlusion and dense crowds partly. Some researchers [24,25] used the symmetric dual-stream network to extract the RGB feature and the depth feature of the image simultaneously. However, it is difficult to acquire the high-quality RGB image feature and depth image feature simultaneously with the symmetric dual-stream network. Specifically, RGB images have rich low-level information (color information, texture information, etc.) and high-level information (face information, body information, etc.), which require a deeper network for extraction. Depth images have mid-level position information (edge shape information, distance information, etc.). If the feature extraction network uses a deeper network, not only will it be more difficult to train the network, but it will also be difficult to retain and extract the useful depth feature. In order to simultaneously acquire the low-level information and high-level information of a high-quality RGB image and the mid-level information of a depth image, it is necessary to design two different feature extraction networks to balance the commonality and characteristics of RGB and depth images.

**Figure 1:**
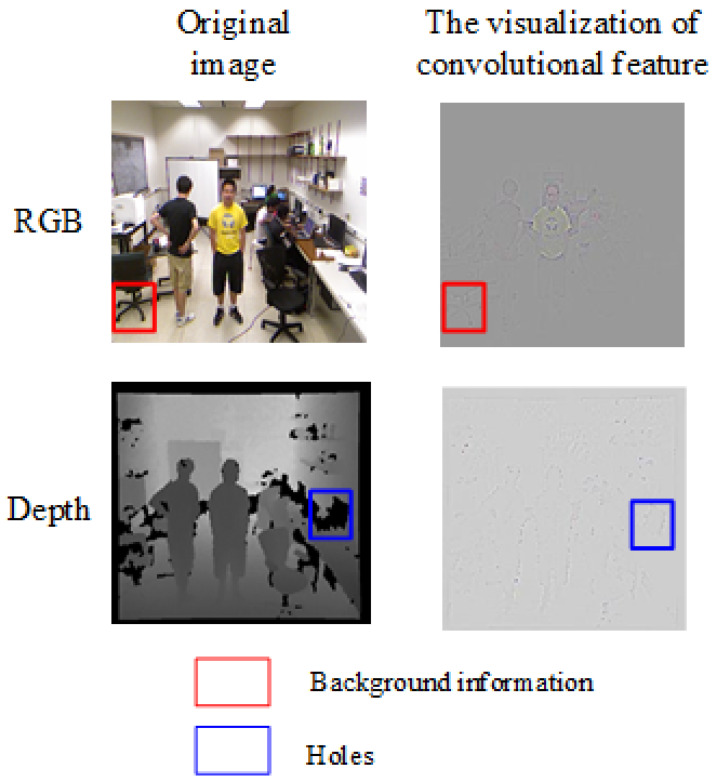
The visualization of the convolutional feature. The red rectangular box indicates the background information. The blue rectangular box indicates the holes

In addition, some background information is included in the RGB feature. The depth feature has some holes due to the depth camera’s sampled images. This will infect the quality of the convolutional feature. Figure 1 shows the visualization of the convolutional feature.

As shown in Figure 1, there are some background information and holes in the RGB feature and the depth feature. If the RGB feature and depth feature are directly stitched or combined, this will increase the redundant background information and empty information, which will affect the quality of the tracking task.

To solve the problems of existing MOT algorithms, we propose a Multi-Object Tracking algorithm for RGB-D images based on Asymmetric Dual Siamese networks (ADSiamMOT-RGBD). This algorithm includes the Trajectory Generation Module (TGM) and the Trajectory Optimization Module (TOM).

There are three main motivations for our algorithm in this paper.

1. In recent years, the accuracy of the single-object tracker in short-term tracking tasks has been greatly improved. Therefore, we transform the MOT task into the multiple short-term single-object tracking task, and we use the high quality of short trajectories to generate the high quality of the target’s trajectories.
2. The RGB images and the depth images contain different information. Currently, the asymmetric feature extraction networks can better consider the characteristics of the RGB image and depth images. In order to obtain a high quality of the RGB-D feature, we design the asymmetric feature extraction network.
3. The MOT task is a strongly time-sequential task. When the targets occlude each other or disappear from the scene, the trajectory association results of the target in the neighboring video subsequences will change accordingly. Therefore, we use the trajectory association results of the neighboring video subsequences to determine the quality of the target trajectory. We optimize the target trajectory according to different qualities to improve the target tracking quality.

In particular, the TGM detects all targets in the scene through RGB images. Subsequently, the asymmetric Siamese tracker module extracts the RGB feature and depth feature of the targets through RGB images and depth images. Later, this module fuses the RGB feature and depth feature through an attention module. This fusion method not only reduces the background information and holes, but also improves the tracking quality of the tracking algorithm. TOM combines the target trajectory fragments of multiple video subsequences with the results of the head detector module. This integrates and optimizes all the target trajectory fragments in the video sequence with the time context information. This method reduces the number of error trajectories that are established due to the false detection results of the head detector module, solves the problem of the frequent conversion of target track ID information, and reduces the number of trajectory interruptions.

There are three main contributions of this paper.

1. To solve the problem that the existing feature extraction networks cannot balance the differences between the RGB feature and depth feature, this paper designs the asymmetric dual Siamese network to balance the information of the RGB feature and depth feature and to extract the high-quality RGB feature and depth feature based on the characteristics of RGB images and depth images.
2. To solve the problem that there is a large amount of redundant information in the fused RGB-D feature, this paper uses an attention mechanism to fuse the RGB feature and depth feature based on the importance of the feature’s location and channel and reduce the redundant information and holes in the RGB-D feature.
3. To solve the problem that the existing MOT algorithm is easy to establish a target track on the wrong target position, this paper designs a trajectory optimization module to analyze the trajectory based on the time context information of the video sequence and suppress the error trajectories to improve the quality of the tracking algorithm.

The structure of this paper is shown as follows. Section 2 describes the related works in the field of MOT. Section 3 describes the proposed algorithm. Section 4 presents the experimental results, and Section 5 presents the conclusions of this paper. Our code will be released at https://github.com/I3-Laboratory/ADSiamMOT.

## 2. Related Work

In recent years, both domestic and international researchers have proposed a large number of MOT algorithms, which can be divided into two categories based on the image categories used, namely the MOT algorithms based on RGB images and the MOT algorithms based on RGB-D images.

### 2.1. The MOT Algorithms Based on RGB Images

These algorithms mainly use the appearance information from RGB images to track all the targets in the scene. In recent years, researchers have proposed a large number of MOT algorithms. Some researchers transformed the MOT task into a data correlation task. They incorporated data correlation algorithms such as the Hungarian algorithm and the KMalgorithm into the trajectory correlation module in the MOT algorithm. Some other researchers transformed the MOT task into multiple Single-Object Tracking (SOT) tasks. They modified the SOT algorithms to improve the quality of the MOT algorithm. In summary, this paper classifies the MOT algorithms into two types based on different solutions for MOT tasks: the MOT algorithms based on data association and the MOT algorithms based on the SOT algorithm.

#### 2.1.1. The Algorithms Based on Data Association

Naiel et al. [7] developed the MOT algorithm for detectors and trackers within a particle filtering framework. They considered each detection region as an important sampling example and used a frame-by-frame data correlation algorithm between the detector and the tracker. Eiselein et al. [8] proposed the MOT algorithm based on Gaussian Mixture Probability Hypothesis Density (GMPHD). Furthermore, this algorithm incorporates the results of the detector to improve the quality of MOT. Bewley A. et al. [9] detected the targets in the scene using the Faster R-CNN object detection algorithm [26]. Then, this algorithm predicted the position of each target using a Kalman filter and correlated the detection results with each trajectory using a Hungarian algorithm based on the target’s motion information to generate target trajectories. In order to improve the ability of target identification, Bewley A. et al. [10] improved their proposed algorithm [9]. They first used a re-identification network to extract the target appearance information. Later, they combined the appearance information with motion information for correlating target trajectories and improving the quality of the MOT algorithm. Bochinski et al. [11] improved the Intersection over Union (IoU) function and proposed the IoU tracker. They used the object detector to detect the targets in the scene. Next, they correlated and generated the trajectories based on the distance between the target and the trajectory. Sheng et al. [12] used GoogLeNet [27] to extract the appearance feature of the target at first. Next, they used the cosine distance of the feature to calculate the degree of similarity between the detection region and the trajectory region. Finally, they optimized all trajectories by the motion information and the degree of similarity.

However, these algorithms depend on the quality of the results of the object detection algorithm too much. If the object detection algorithm misses or wrongly checks the target location in the scene, the trajectory will be greatly affected, and this will result in the tracking problem of the number of ID switches, which makes it difficult to obtain the complete trajectory of the target. Moreover, such algorithms are difficult to correctly track and identify humans with a similar appearance and close distance in the scene, resulting in trajectory drift.

#### 2.1.2. The Algorithms Based on the SOT Algorithm

Comaniciu et al. [13] applied the mean shift algorithm to the MOT task. They first predicted the target position in the current scene. Then, they used the Bhattacharyya coefficient to calculate the apparent similarity between the target and the candidate object. Finally, their algorithm predicted the position of the target based on the apparent similarity and output the complete trajectory. Avitzour [14] and Gordon [15] were the pioneers in applying the particle filter algorithm to the MOT task, which was subsequently improved by numerous researchers. Daneseu et al. [16] set a global particle filter and multiple local particle filters to estimate each target position in the scene and finally combined the results of the global particle filter and local particle filter to output the trajectory.

With the development of deep learning and SOT techniques, some researchers have adopted the Siamese SOT algorithm for the MOT task. Junbo et al. [17] proposed the MOT algorithm, UMA, which tracked each target in the scene via the SiamFCtracker and subsequently used the appearance feature, which was extracted by SiamFC to associate trajectories. Their algorithm reduced the computation time of feature extraction. Feng et al. [18] used the SiamRPNtracker to obtain the short-term trajectory of each target. Later, they used the re-identification network to extract the appearance feature of the target and calculated the matching confidence between the target and the trajectory. Finally, their algorithm associated multiple short-term trajectories to generate complete trajectories based on the matching confidence, which solved the problem of trajectory drift. Peng et al. [19] first used the Siamese-style network to extract the target feature and track all the targets in the scene. After, their algorithm obtained many of the short-term trajectories of the targets. Subsequently, their algorithm used the features that were extracted from the Siamese-style network to calculate the similarity between the target and numerous short-term trajectories. To generate the correct trajectories, they utilized the R1TApower iteration layer [28] to generate the trajectories. Zhu et al. [20] improved the SOT tracker ECO [29] and used the improved ECO tracker to track all targets in the scene. They also used the Bi-LSTM network [30] to extract the feature of the target and analyzed the historical feature of the target for correlating and optimizing the trajectory.

However, these algorithms did not judge the correctness of the detection results. When the target detection module incorrectly outputs the results, these MOT algorithms are prone to establish target trajectories at the wrong target locations. These algorithms will generate a large number of wrong trajectories and reduce the quality of the trajectory.

### 2.2. The MOT Algorithms Based on the RGB-D Images

The MOT algorithms based on RGB-D images extract appearance information and Three-Dimensional (3D) distance information from RGB and depth images, then use the appearance information and 3D distance information to detect all the targets in the scene. Finally, these algorithms use the similarity of the target’s appearance or the target’s movement information to correlate and generate the trajectory.

Chrapek et al. [31] extended the RGB tracker, TLD [32] (Tracking-Learning-Detection), to depth sequences. They used the depth image as an additional feature in the tracking phase to improve the feature quality. Meanwhile, they computed the mean depth change of the target to determine the target’s occlusion state and scale information. Later, they used the occlusion state and scale information to improve the results of their algorithm. Qi et al. [33] combined optical flow information, color information, and depth information and proposed a multi-cue MOT framework. They computed the optical flow information of the target on RGB images and approximately estimated the motion information of the target based on the optical flow information at first. Then, they divided the target area into four subregions (top, bottom, left, and right) and computed the color histogram distribution feature and the mean depth information of the target in each subregion. Subsequently, the trajectories were correlated according to the color histogram distribution feature and the mean depth information of the target. Liu et al. [34,35,36] used the target appearance color histogram feature and depth histogram information to detect all targets in the scene from RGB-D images. Next, they created the trajectory for each target, which was correlated by the apparent similarities in target and trajectory. Ma et al. [37] used the HOG feature-based DPM object detection algorithm [38] to detect the target in RGB and depth images. Then, they used the conditional random field-based approach [39] to work out the data correlation task and the trajectory estimation task. Li et al. [40] provided a multi-object tracking algorithm based on the RGB-D data. Firstly, they used the YOLOv2 object detection algorithm to detect the targets frame-by-frame. Then, it output the trajectories by the correlation algorithm, which was based on the characteristics of the target’s depth histogram distribution, the IoU, and Euclidean distance between the detection results in the neighboring frames.

However, the above algorithms only use depth images to extract the low-level feature (edges, texture information, etc.) of the target and do not fully extract the high-level feature of the depth images. Moreover, the depth feature extracted by the above algorithms still contains void regions in the depth image, which makes it difficult to extract a high-quality RGB-D feature to identify the target in the scene. In other words, it restricts the discrimination ability of the MOT algorithm.

## 3. The Proposed Algorithm

### 3.1. The Overall Structure of the Algorithm

**Figure 2:**
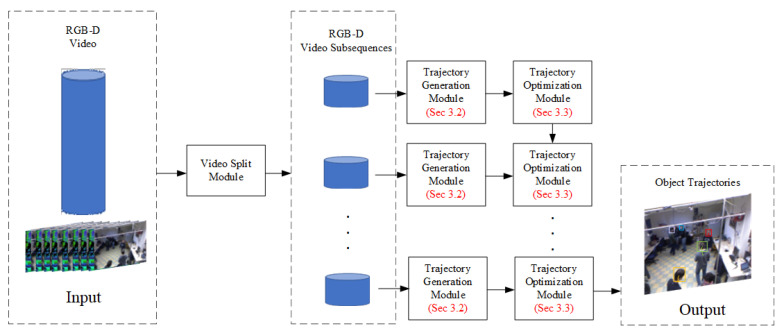
The overall flowchart of the tracking algorithm. The Trajectory Generation Module (TGM) will be introduced in Section 3.2; the Trajectory Optimization Module (TOM) will be introduced in Section 3.3.

In order to solve the problems of existing MOT algorithms, we propose a tracking algorithm based on RGB-D images. This algorithm mainly includes a video slicing module, a Trajectory Generation Module (TGM), and a Trajectory Optimization Module (TOM). Figure 2 shows the overall flow chart of the tracking algorithm.

As shown in Figure 2, the proposed algorithm firstly slices the video sequence into several video subsequences at regular time intervals by the video slicing module. Then, the video sequence is input to the TGM to track all the targets. Finally, several trajectories are optimized according to the time context information by the TOM.

### 3.2. The Trajectory Generation Module

**Figure 3:**
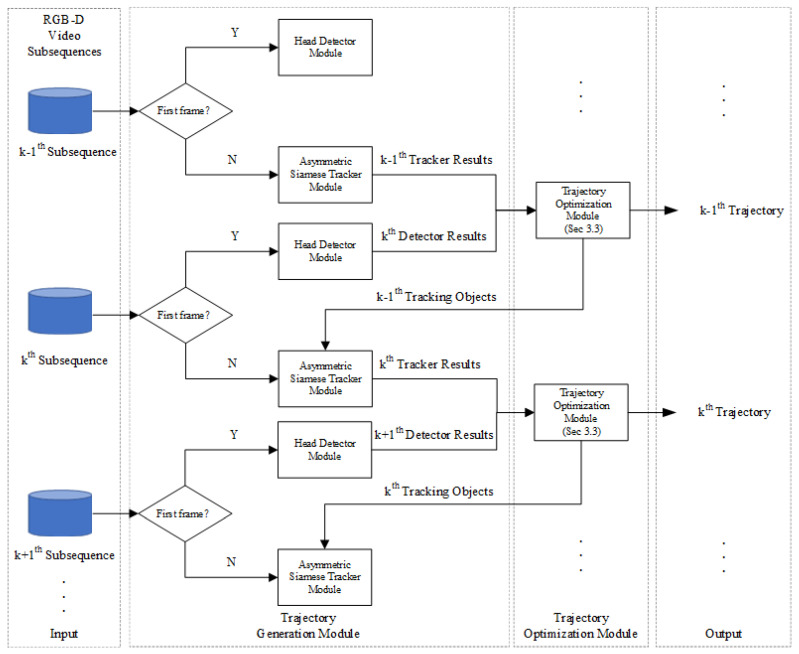
The flowchart of Trajectory Generation Module (TGM).

In order to solve the problem that the existing MOT algorithm cannot balance the differences between the RGB feature and depth feature and the problem that there is much redundant information in the RGB-D feature, we propose a Trajectory Generation Module (TGM), which consists of the head detection module and the asymmetric Siamese Tracker module, and its flowchart is shown in Figure 3.

In particular, the head detection module consists of the YOLOv3 [41] object detection algorithm. In this paper, the YOLOv3 object detection algorithm is trained by the human head dataset (RGB images only) so that it can detect the human head based on RGB images. In the testing phase, the detector results from the head detector module are first input to the Trajectory Optimization Module (TOM) to determine the correct tracking target. Then, the TOM inputs the tracking objects into the asymmetric Siamese tracker module as the template branch input.

#### 3.2.1. The Characteristics of RGB Images and Depth Images

**Figure 4:**
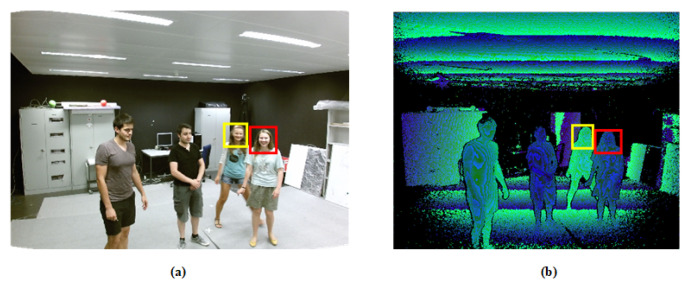
The RGB image and the depth image at the same time of the video sequence. (**a**) RGB image. (**b**) Depth image.

In RGB images, the heads of different humans often have a similar appearance. In depth images, two human heads with close positions have different distance and edge contour information, which is useful for distinguishing the identity of humans with a close appearance or motion information. Figure 4 shows the RGB image and depth image of the video sequence at the same moment. In this paper, we mark the head of the red box as Person A and the head of the yellow box as Person B.

As shown in Figure 4, in the RGB image, Person A and Person B have similar facial, hair color, and another head appearance feature. Furthermore, the locations of Person A and Person B are close, and there is little difference in the motion information of Person A and Person B, which is calculated in the time-series contextual information from the RGB image. The depth image reflects directly the distance information of Person A and Person B from the scene. However, the depth image does not contain appearance information about the human head, and it is impossible to mark the target’s identity information.

#### 3.2.2. The Design of the Asymmetric Siamese Tracker Module

**Figure 5:**
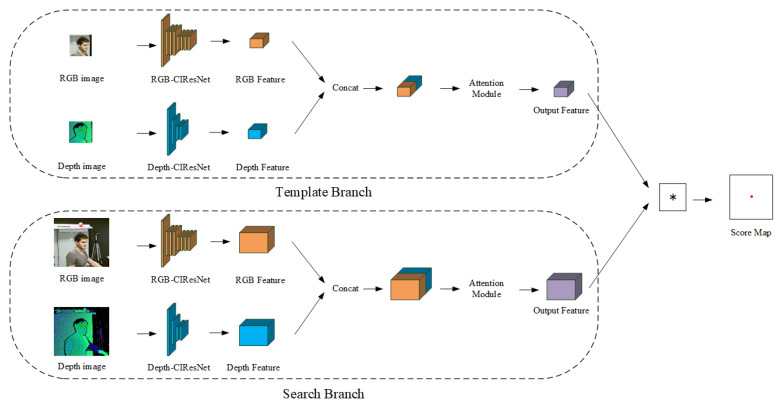
The network structure of the asymmetric Siamese tracker module. The module consists of a template branch, a search branch, and a cross-correlation response branch. * represents the cross-correlation convolution operation.

The RGB image and the depth image contain different information, where the RGB image contains the rich head appearance feature and the depth image contains the robust 3D position information. According to the characteristic of the RGB and depth image, we design the asymmetric Siamese tracker module based on the the SiamFC [42] tracker and the CIResNetnetwork structure [43] to acquire the high-quality feature from the RGB image and the depth image. Meanwhile, we import the attention module to reduce the redundant information from the RGB-D feature. The network structure of the asymmetric Siamese tracker module is shown in Figure 5.

As can be seen in Figure 5, the network structure of the asymmetric Siamese tracker module consists of two branches, the template branch and the search branch. First, we use the template branch and the search branch to extract the RGB-D feature of the target and search region, respectively. Next, we compute the similarity score of the target and search areas by the cross-correlation operation. Finally, we determine the location of the target in the search area based on the similarity score.

(1) The design of the asymmetric dual Siamese network:

Based on the characteristics of the RGB image and the depth image, we design the RGB feature extraction network (RGB-CIResNet) and the depth feature extraction network (Depth-CIResNet), respectively. The RGB-CIResNet network structure is consistent with the CIResNet-22 [43] fully convolutional neural network structure. Compared to the RGB-CIResNet network, the Depth-CIResNet network crops some residual blocks. The network structures of the RGB-CIResNet and the Depth-CIResNet are shown in Table 1.

**Figure 6:**
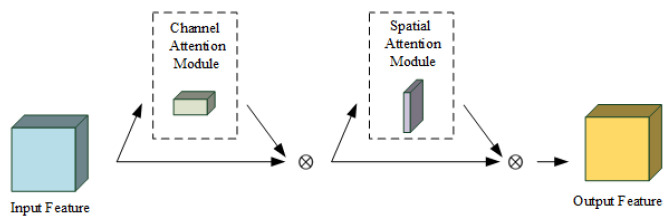
The structure of the CBAMattention module.

(2) The design of the RGB-D feature fusion algorithm:

In recent years, the attention module [44] in deep learning has received much attention as a method to improve feature quality. The attention module enhances the feature that is most helpful to the task. The common SE-Net [45] attention module focuses only on the weight relationship between feature channels. Compared to it, the CBAM [46] attention module integrates the weight relationship between feature channels and spatial information. The structure of the CBAM is shown in Figure 6.

As can be seen in Figure 6, the CBAM attention module first feeds the RGB-D feature into the channel attention module to extract the channel weight relationship of the RGB-D feature. Then, the channel weight relationship is weighted and fused with the input feature to output the channel attention feature $M_{c}\left(F\right)$. The calculation of the channel attention module is shown in Formula (1).

(1) $$\begin{array}{cc} M_{c}\left(F\right) & =\sigma \left(MLP\left(AvgPool\left(F\right))+MLP(MaxPool\left(F\right)\right)\right)\\ \; & =\sigma \left(\left(MLP\left(F_{avg}^{c}\right)\right)+\left(MLP\left(F_{max}^{c}\right)\right)\right) \end{array}$$

where *F* represents the input feature. $F_{avg}^{c}$ and ${\;F}_{max}^{c}$ represent the AvgPool feature and the MaxPool feature generated by the input feature F by the AvgPool and the MaxPool operation, respectively. MLP represents the Multilayer Perceptron. σ represents the sigmoid operation, and $M_{c}\left(F\right)$ represents the output feature processed by the channel attention module. Next, the channel attention feature $M_{c}\left(F\right)$ is fed into the spatial attention module to extract its spatial weighting relationship. Later, The spatial weighting relationship is weighted and fused with the channel attention feature $M_{c}\left(F\right)$ to output the spatial attention feature $M_{s}\left(F\right)$. The calculation of the spatial attention module is shown in Formula (2).

(2) $$\begin{array}{cc} M_{s}\left(F\right) & =\sigma \left(f^{7*7}\left(\left[AvgPool\left(F\right);MaxPool\left(F\right)\right]\right)\right)\\ \; & =\sigma \left(f^{7*7}\left(\left[F_{avg}^{S};F_{max}^{S}\right]\right)\right)\\ \; & =\sigma \left(f^{7*7}\left(F_{concat}^{S}\right)\right) \end{array}$$

where $F_{avg}^{s}$ and $F_{max}^{s}$ represent the AvgPool feature and the MaxPool feature generated by the channel attention feature $M_{c}\left(F\right)$ by the AvgPool and the MaxPool operation, respectively. $F_{concat}^{S}$ represents the feature acquired after $F_{avg}^{s}$ and $F_{max}^{s}$ perform the concat operation. σ represents the sigmoid operation. $f^{7*7}$ represents the convolution operation. The size of the convolutional core is 7∗7. $M_{s}\left(F\right)$ represents the output feature processed by the spatial attention module.

(3) The design of the cross-correlation operation:

According to the translation invariance of the feature, which is obtained by the fully convolutional neural network, we use the RGB-D feature φ(z) of the template branch as the convolution kernel. Then, we perform the cross-correlation operation between the convolution kernel φ(z) and the RGB-D feature φ(x) of the search branch to obtain the score response map of the target position. The calculation of the cross-correlation operation is shown in Formula (3).

(3) $$\begin{array}{c} f\left(z,x\right)=\varphi \left(z\right)*\varphi \left(x\right) \end{array}$$

where f(z,x) represents the target position score response matrix and ∗ represents the convolution operation. The asymmetric Siamese tracker module determines the position of the target in the search area based on the maximum value of the score response matrix.

### 3.3. The Trajectory Optimization Module

To address the problem that existing MOT algorithms are prone to establish target trajectories at the wrong target locations, we design a Trajectory Optimization Module (TOM). This module consists of the trajectory correlation module and the trajectory analysis module.

**Figure 7:**
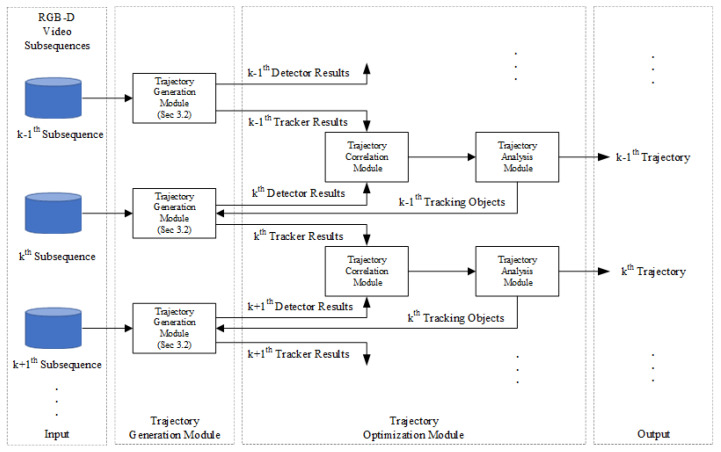
The flowchart of Trajectory Optimization Module (TOM).

As shown in Figure 7, firstly, the trajectories of the k−1th video subsequence and the head detection results of the *k*th video subsequence will be input to the trajectory correlation module to correlate the head movement trajectories of the two neighboring video subsequences. Subsequently, the trajectory analysis module uses the trajectory information that is output by the trajectory correlation module to optimize the head movement trajectories. In particular, the trajectory correlation module is mainly composed of the Hungarian algorithm [47].

#### 3.3.1. The Characteristics of the Trajectory

**Figure 8:**
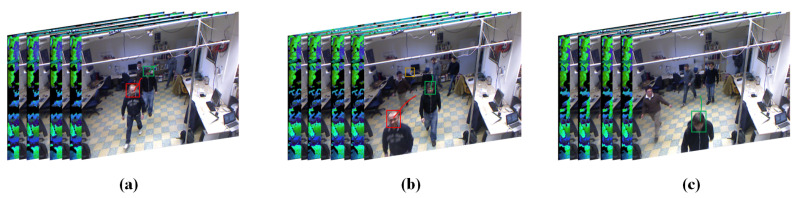
Some head movement trajectories in some video subsequences. (**a**) k−1th subsequence. (**b**) *k*th subsequence. (**c**) k+1th subsequence.

In video sequences, the head movement trajectories are often influenced by the results of the head detector module. TGM is prone to generate the wrong head movement trajectories when the head detector module gives the wrong results due to the complex background or occlusion from other people. Figure 8 shows some head movement trajectories in some video subsequences.

As shown in Figure 8, there are four types of head movement trajectories in each video subsequence: (1) correct head movement trajectories (green box); (2) interrupted head movement trajectories (blue box); (3) incorrect head movement trajectories (yellow box); and (4) disappearing head movement trajectories (red box).

The interrupted head movement trajectory is generated due to the head detection module missing the head in the scene, and such trajectories increase the time of ID switching error and the number of the missed trajectory of the MOT algorithm. The incorrect head movement trajectory is generated due to the head detection module detecting other objects in the scene, and such a trajectory increases the time of ID switching error and the number of the false trajectories of the MOT algorithm. In summary, the wrong results of the head detection module will reduce the quality of the trajectory.

#### 3.3.2. The Design of the Trajectory Optimization Module

In order to reduce the interrupted and incorrect head movement trajectories that are output by the target trajectory generation module, we design a trajectory analysis module based on the contextual information of video sequences. This module determines the trajectory category based on the head detection results of neighboring video sequences and the position of each head trajectory. Then, this module adjusts the trajectory generation module according to the trajectory category.

The trajectory analysis module classifies the four types of head movement trajectories into three categories. (1) High-quality trajectory: This class of trajectories can completely cover the head movement trajectories. These trajectories are composed of correct head movement trajectories. (2) Low-quality trajectory: These trajectories fail to cover all the trajectories because the head detection module misses the head targets in the scene. These trajectories are composed of interrupted head movement trajectories. (3) Error/disappeared trajectory: These trajectories should be deleted from the collection of head movement trajectories due to people moving out of the scene or the head detection module outputting the wrong results. Such trajectories are composed of the incorrect head movement trajectory and disappearing head movement trajectory.

In particular, the high-quality trajectory can obtain a successful correlation result in the trajectory correlation module of each video subsequence. The low-quality trajectory often fails to obtain a successful correlation result in the trajectory correlation module of a video subsequence because the head detection module misses the head target in the scene. The error/disappeared trajectory fails to obtain a successful correlation result in the trajectory correlation module in neighboring video sequences because the people move out of the scene or the head detection module outputs the wrong results. Based on the different characteristics of the above three categories of the trajectory, we design a trajectory analysis module based on the correlation results of the trajectory correlation module.

**Figure 9:**
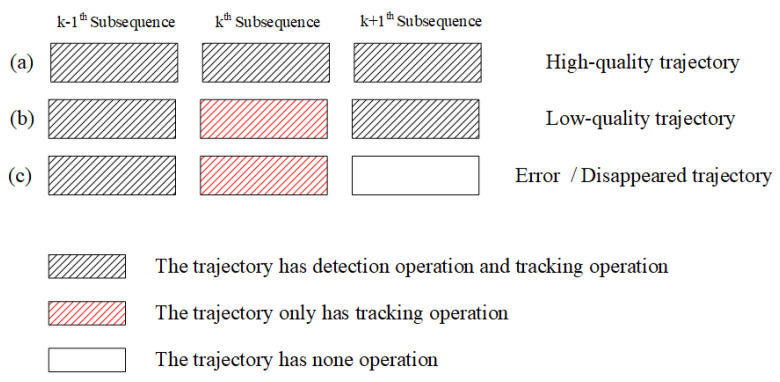
Corresponding trajectory generation strategy for each type of trajectory.

According to the results of the trajectory analysis module, the corresponding trajectory generation strategies for each category of trajectories and those strategies are shown in Figure 9.

From Figure 9, in each video subsequence, high-quality trajectories are used in the trajectory generation module for head detection and tracking operations to update the head information and trajectory.

When the head detection module misses the head target in the kth video subsequence, the trajectory generation module only carries out the tracking operation. At this time, the trajectory generation module can still provide a relatively accurate trajectory of the head target to reduce the low-quality trajectories caused by the missing detection of the head detection module.

When the head detection module fails to detect other objects or wrong head targets in *k*th and k+1th video subsequences, the error/disappeared trajectory will be deleted from the set of trajectories to save the computation time of the MOT algorithm.

## 4. Experiments

This section presents the experimental results and analysis of the ADSiamMOT-RGBD algorithm. Section 4.1 describes the computer environment and the datasets for the experimental test. At the same time, we describe the MOT evaluation metrics. Section 4.2 describes the ablation experiments to validate the effectiveness of the trajectory generation module and the trajectory optimization module. Section 4.3 shows the tracking results of the ADSiamMOT-RGBD algorithm and the state-of-the-art MOT algorithms for validating the advantages of the ADSiamMOT-RGBD algorithm.

### 4.1. Experiment Details

Our experiments were implemented using the pytorch framework on a computer with a GTX1660Ti graphics card. The datasets used in this paper include three datasets: MICC [48], EPFL [49], and UM [50].

The MICC [48] dataset was filmed in the laboratory with an RGB-D camera. It includes three scenes: Flow, Group, and Queue. Specifically, the Flow scene is a scene of people moving forward and backward. The Group scene is a scene of people gathering and moving, and the Queue scene is a scene of people moving in a queue.

The EPFL [49] dataset was filmed in the laboratory and indoor corridors with an RGB-D camera. It includes two scenes: EPFL-LAB and EPFL-CORRIDOR. Both scenes contain varying degrees of pedestrian occlusion and scale variation. These scenes are very challenging for MOT algorithms.

The UM [50] dataset was filmed in the laboratory with an RGB-D camera. This dataset has fewer situations of people occlusion.

We used the evaluation metrics proposed in the MOT Challenge [51] to evaluate our experiments. In the MOT evaluation metrics, the MOTAmetric and the MOTPmetric are the two main evaluation metrics.

The MOTA metric concerns the tracking accuracy of the tracker. This metric is related to three sources of errors FP, FN, and IDSin the tracking. The more errors that occur in the tracking algorithm, the lower the MOTA metric. The calculation process is shown in Formula (4).

(4) $$\begin{array}{c} MOTA=1-\frac{\Sigma \left(FN+FP+IDS\right)}{\Sigma GT} \end{array}$$

where FP is a false positive that describes an unannotated target. FN is a false negative that describes a missed target. IDS is the number of target ID switches. GT is the number of ground truth objects.

The MOTP metric is the average dissimilarity between all true positives and their corresponding ground truth targets. This metric represents the degree of mismatch between the annotation box and the prediction box. The calculation process is shown in Formula (5).

(5) $$\begin{array}{c} MOTP=\frac{\Sigma_{t,i}d_{t,i}}{\Sigma_{t}c_{t}} \end{array}$$

where $c_{t}$ denotes the number of matches in frame t and $d_{t,i}$ is the bounding box overlap of target *i* with its assigned ground-truth.

In addition, there are three other metrics that need to be presented in this paper, they are FM, MT, and ML: FM represents the total number of times a trajectory is fragmented. MT represents the Mostly Tracked targets; this means that the ratio of ground-truth trajectories that are covered by a track hypothesis for at least 80% of their respective lifespan. ML represents the Mostly Lost targets; this means that the ratio of ground-truth trajectories that are covered by a track hypothesis for at most 20% of their respective lifespan.

### 4.2. Ablation Study

We designed two ablation experiments to verify the effectiveness of the trajectory generation module and the trajectory optimization module. These two ablation experiments validate the trajectory generation module proposed in Section 3.2 and the trajectory optimization proposed in Section 3.3, respectively.

#### 4.2.1. The Effectiveness of the Trajectory Generation Module

To verify the effectiveness of the trajectory generation module, the experiment used the same head detector module and trajectory optimization module. In addition, the experiment only changed the Siamese network structure in the trajectory generation module to control the experimental variables. The experiment denotes two Siamese network structures. They are the original Siamese network and the asymmetric dual Siamese network, respectively. In order to distinguish the above network structures, the experiment denotes the algorithm that used the original Siamese network as ADSiamMOT-RGB and the algorithm that used the Asymmetric Dual Siamese network as ADSiamMOT-RGBD. The experiment evaluates both networks on MICC, EPFL, and UM. The results are shown in Table 2.

According to Table 2, it can be seen that the MOTA metric of the ADSiamMOT-RGBD algorithm is superior to the ADSiamMOT-RGB algorithm on the three datasets.

Specifically, on the MICC and EPFl datasets with high levels of occlusion, compared to the original Siamese network structure, the MOTA metrics of the asymmetric Siamese tracker module improve by 3.7% and 7.3%, respectively, on the MICC and EPFL datasets with more occlusion. The large decreases in the FP, FN, and IDS metrics indicate that the asymmetric Siamese tracker module reduces the number of tracking errors. These experimental results demonstrate that the asymmetric Siamese tracker module has better tracking accuracy and stability than the original Siamese network structure.

On the UM datasets with less occlusion, compared to the original Siamese network structure, the MOTA matrices of the asymmetric Siamese tracker module improve by 8.1%. Although there is no significant difference between the FP and IDS metrics of two Siamese network structures, the FN metric declines significantly. This result indicates that the asymmetric Siamese tracker module can effectively reduce the number of missed detections and improve the tracking accuracy when there are fewer occlusions in the tracking scene.

In summary, the MOTA metric of the asymmetric Siamese tracker module outperforms that of the original Siamese network structure on all three datasets. This demonstrates the effectiveness of the trajectory generation module that uses the asymmetric dual Siamese network.

#### 4.2.2. The Effectiveness of the Trajectory Optimization Module

To verify the effectiveness of the trajectory optimization module, we select different time intervals for testing. Specifically, we use the same head detector module and the trajectory generation module to control the experimental variables. When the interval is zero, the trajectory optimization module is not used in the tracking task. The larger the time interval, the longer the subsequence. We tested the algorithms on three datasets, MICC, EPFL, and UM. The test results are shown in Table 3.

According to Table 3, it can be seen that the MOTA metric of the trajectory optimization module is superior to the unused trajectory optimization module on all three datasets. Specifically, compared to the unused trajectory optimization module, the MOTA metric of the trajectory optimization module improves by 5.6%, 1.5%, and 2.2%, respectively, on the MICC, EPFL, and UM datasets, and the IDS metric decreases by 107, 21, and 301, respectively. This shows that the trajectory optimization module effectively reduces the number of IDS, indicating that it can effectively solve the problem of disconnected target trajectories and improve the tracking accuracy and stability of the MOT algorithm. Furthermore, with respect to the trajectory optimization module itself, as the time interval increases, the IDS metric decreases, and the FN metric increases. This is because the same target is tracked steadily over a certain time interval, reducing the number of IDS. However, when a new target appears, it cannot be tracked along that time interval. This would result in missed detections and an increase in the FN metric. Therefore, the time interval needs to be reduced appropriately when there are more new targets in the video sequence. The time interval can be increased appropriately when there are fewer new targets.

In summary, it is shown that the trajectory optimization module is useful for improving tracking accuracy and stability.

### 4.3. State-Of-The-Art Comparison

To verify the effectiveness of the ADSiamMOT-RGBD algorithm, we selected many algorithms for comparison. These algorithms include Sort [9], DeepSort [10], IoU-tracker [11], SST [52] and the ADSiamMOT-RGB algorithm. We tested the algorithms on three datasets, MICC, EPFL, and UM. The test results are shown in Table 4.

According to Table 4, it can be seen that the MOTA metric of the ADSiamMOT-RGBD outperforms each comparison tracking algorithm on all three datasets. We rank the MOTA metric for all comparison tracking algorithms from highest to lowest. Red, green, and blue represent 1st, 2nd, and 3rd, respectively.

On the MICC dataset, the top three algorithms are the ADSiamMOT-RGBD algorithm, the ADSiamMOT-RGB algorithm, and the Sort algorithm. On the EPFL dataset, the top three algorithms are the ADSiamMOT-RGBD algorithm, the ADSiamMOT-RGB algorithm, and the IoU-tracker algorithm. On the UM dataset, the top three algorithms are the ADSiamMOT-RGBD algorithm, the ADSiamMOT-RGB algorithm, and the Sort algorithm.

Specifically, compared to the ADSiamMOT-RGB algorithm and the Sort algorithm, the MOTA metric of the ADSiamMOT-RGBD algorithm improves by 3.1% and 5.3%, respectively, on the MICC dataset. The decreases in the IDS and FN metric indicate that the ADSiamMOT-RGBD algorithm is effective at reducing the number of target ID switches and target misses. It demonstrates the effectiveness of the ADSiamMOT-RGBD algorithm for improving tracking accuracy and stability. Compared to the ADSiamMOT-RGB algorithm and the IoU-tracker algorithm, the MOTA metric of the ADSiamMOT-RGBD algorithm improves by 0.4% and 13.3%, respectively, on the EPFL dataset. The decrease in the FN metric indicates that the ADSiamMOT-RGBD algorithm is useful for improving the accuracy and stability of tracking. On the UM dataset, compared to the ADSiamMOT-RGB algorithm, the ADSiamMOT-RGBD algorithm has almost an equal MOTA metric and IDS metric. Compared to the Sort algorithm, the MOTA metric for the ADSiamMOT-RGBD algorithm improves by 1.8%, for which there is a significant decrease in the FN metric.

In summary, the ADSiamMOT-RGBD algorithm achieves more competitive results on all three datasets. It shows that the ADSiamMOT-RGBD algorithm can effectively improve tracking accuracy and stability.

### 4.4. The Discussion of the Time Consumption

In the training phase, our algorithm requires 19.17 h for training the head detector module and 1.67 h for training the asymmetric Siamese tracker module.

In the testing phase, we computed the time consumption of each algorithm, which was mentioned in Section 4.3. The time consumption of each algorithm is shown in Table 5.

As shown in Table 5, for IoU-tracker, Sort, and DeepSort, they belong to the algorithm based on the data association. Such algorithms use the real-time object detector and the correlation algorithm with a small computational cost to achieve MOT tasks. Because of the real-time object detector and the correlation algorithm with a small computational cost, these algorithms have high speed on the MOT task. As shown in Figure 5, although these algorithms have high FPS, their MOTA metrics are lower. The reason is that these algorithms rely heavily on the quality of the object detector and are prone to trajectory interruption problems.

For our algorithms (including ADSiamMOT-RGB and ADSiamMOT-RGBD), they have great MOTA metrics for the MICC, EPFL, and UM datasets. However, our algorithms have a low speed on the MOT task. The reason is that our algorithms need to build up many single-object trackers for each target and need numerous computations to obtain the target’s RGB or RGB-D features. In the future, we will modify the feature extraction network and reduce the computation of the feature extraction.

## 5. Conclusions

In this paper, we propose the ADSiamMOT-RGBD algorithm. The algorithm includes the trajectory generation module and the trajectory optimization module. Specifically, the trajectory generation module extracts the RGB feature and depth feature by the asymmetric Siamese network, then it fuses the RGB feature and depth feature to form a high-quality RGB-D feature by the attention mechanism. the trajectory optimization module suppresses error trajectories based on the video sequence’s contextual information. The experiments show that the ADSiamMOT-RGBD algorithm solves the target’s trajectory drift problem and ID interruption problems, and it achieves competitive results on three datasets MICC, EPFL, and UM. In the future, we will optimize the computation of the asymmetric Siamese tracker module to improve the speed of tracking and infuse depth images in the head detector module to improve the robustness of the algorithm.

## Figures and Tables

**Table 1:** The network structure of the RGB-CIResNet and the Depth-CIResNet.

Stage	RGB-CIResNet	Depth-CIResNet
Conv1	7×7, 64, stride 2	
Conv2	2×2, max pooling, stride 2	
	$\left[\begin{array}{cc} 1\times 1 & 64\\ 3\times 3 & 64\\ 1\times 1 & 256 \end{array}\right]\times$ 3	$\left[\begin{array}{cc} 1\times 1 & 64\\ 3\times 3 & 64\\ 1\times 1 & 256 \end{array}\right]\times$ 1
Conv3	$\left[\begin{array}{cc} 1\times 1 & 128\\ 3\times 3 & 128\\ 1\times 1 & 512 \end{array}\right]\times$ 4	$\left[\begin{array}{cc} 1\times 1 & 128\\ 3\times 3 & 128\\ 1\times 1 & 512 \end{array}\right]\times 2$

**Table 2:** The results of the Asymmetric Dual Siamese Multi-Object Tracking (ADSiamMOT)-RGB and ADSiamMOT-RGBD on each dataset. MT, Mostly Tracked; ML, Mostly Lost.

Dataset	Algorithm	MOTA ↑	MOTP ↑	FP ↓	FN ↓	IDS ↓	FM ↓	MT ↑	ML ↓
MICC	ADSiamMOT-RGB	59.9	69.6	2271	2775	34	225	11	0
	ADSiamMOT-RGBD	**62.1**	69.9	2249	2538	17	269	12	0
EPFL	ADSiamMOT-RGB	39.9	74.7	606	2114	28	61	6	1
	ADSiamMOT-RGBD	**42.8**	74.8	581	2015	19	55	6	1
UM	ADSiamMOT-RGB	66.4	71.7	1903	11,137	39	217	6	1
	ADSiamMOT-RGBD	**71.8**	71.8	1985	9166	42	242	9	1

**Table 3:** Tracking results for different time intervals for the ADSiamMOT-RGBD.

Dataset	Interval	MOTA ↑	MOTP ↑	FP ↓	FN ↓	IDS ↓	FM ↓	MT ↑	ML ↓
MICC	0	60.6	**70.0**	2262	2605	125	321	12	0
	1	61.4	69.9	2339	2502	47	354	13	0
	2	61.8	69.9	2299	2505	33	318	12	0
	3	62.3	69.9	2230	2517	24	275	13	0
	4	63.7	69.8	2151	**2430**	**12**	263	12	0
	5	63.7	69.8	2151	**2430**	**12**	263	13	0
	6	62.6	69.9	2181	2528	22	259	12	0
	7	62.1	69.9	2249	2538	17	269	12	0
	8	62.6	69.6	2150	2559	23	**226**	12	0
	9	**64.2**	69.8	**2037**	2477	18	227	12	0
	10	62.8	69.7	2150	2541	17	250	12	0
EPFL	0	46.7	76.2	546	1834	59	83	11	0
	1	**47.4**	**76.2**	564	**1805**	38	87	11	0
	2	46.6	75.9	566	1846	30	82	10	1
	3	46.9	75.5	**542**	1866	21	77	12	1
	4	45.4	75.6	560	1913	23	74	8	1
	5	45.6	75.2	543	1921	23	69	7	1
	6	42.8	75.1	566	2019	30	69	7	1
	7	42.8	74.8	581	2015	19	55	6	1
	8	42.1	75.1	576	2053	18	58	6	1
	9	42.4	74.6	584	2035	16	60	7	1
	10	39.3	75.3	614	2147	**15**	**53**	4	1
UM	0	70.2	72.1	2077	9397	343	507	9	1
	1	71.4	72.1	2195	**9019**	124	650	9	1
	2	71.6	72.4	2133	9049	83	419	9	1
	3	71.7	72.6	2105	9061	66	355	9	1
	4	71.7	72.6	2106	9076	57	336	9	1
	5	71.4	72.7	2110	9184	48	296	9	1
	6	71.4	72.8	2135	9169	44	276	9	1
	7	**71.8**	71.8	**1985**	9166	42	242	9	1
	8	71.6	72.7	2012	9206	39	240	9	1
	9	71.4	**72.9**	2030	9292	37	249	9	1
	10	70.9	**72.9**	2102	9406	**34**	**235**	9	1

**Table 4:** Tracking results of different algorithms on each dataset.

Dataset	Algorithm	MOTA ↑	MOTP ↑	FP ↓	FN ↓	IDS ↓	FM ↓	MT ↑	ML ↓	Rank ↓	FPS ↑
MICC	Sort	60.8	70.0	1997	2877	84	282	11	0	3	20.98
	DeepSort	59.6	69.2	2212	2874	25	340	11	0	4	13.08
	IoU-tracker	54.0	70.2	1464	4022	336	462	7	0	6	59.16
	SST	55.2	69.5	1633	3958	86	614	6	0	5	2.92
	ADSiamMOT-RGB	62.2	69.4	2006	2744	30	193	10	0	2	2.72
	ADSiamMOT-RGBD	64.2	69.8	2037	2477	18	227	11	0	1	2.64
EPFL	Sort	40.9	76.1	407	2207	87	109	5	1	5	26.48
	DeepSort	41.0	76.4	206	2468	22	112	2	1	4	19.21
	IoU-tracker	41.1	74.9	244	2349	99	120	2	1	3	24.12
	SST	37.9	72.5	289	2480	71	179	4	0	6	2.86
	ADSiamMOT-RGB	47.2	76.2	565	1807	42	91	11	0	2	6.65
	ADSiamMOT-RGBD	47.4	76.2	564	1805	38	87	11	0	1	5.93
UM	Sort	70.5	7.2	1942	9731	41	366	9	1	2	21.91
	DeepSort	67.4	71.9	1444	11,452	59	556	7	1	3	16.69
	IoU-tracker	49.1	75.1	941	18,700	558	658	4	4	5	64.60
	SST	51.9	74.4	1219	17,648	225	1446	4	3	4	3.13
	ADSiamMOT-RGB	71.8	72.2	1992	9173	39	235	9	1	1	7.05
	ADSiamMOT-RGBD	71.8	71.8	1985	9166	42	242	9	1	1	5.52

**Table 5:** The time consumption of each algorithm.

Algorithm	Dataset	FPS ↑	Average FPS ↑	Average MOTA ↑
Sort	MICC	20.98	23.12	57.40
	EPFL	26.48		
	UM	21.91		
DeepSort	MICC	13.08	16.33	56.00
	EPFL	19.21		
	UM	16.69		
IoU-tracker	MICC	59.16	49.29	48.06
	EPFL	24.12		
	UM	64.60		
SST	MICC	2.92	2.97	48.33
	EPFL	2.86		
	UM	3.13		
ADSiamMOT-RGB	MICC	2.72	5.47	60.40
	EPFL	6.65		
	UM	7.05		
ADSiamMOT-RGBD	MICC	2.64	4.70	61.13
	EPFL	5.93		
	UM	5.52		
